# Evaluating Drug‐Target Complexes in Therapeutic Monoclonal Antibodies Using A4F‐MALS: Implications for Drug Development and Clinical Design

**DOI:** 10.1155/jimr/8063033

**Published:** 2026-05-29

**Authors:** Nina Liu, Donald Kotowski, Eric T. Ong, Kishor B. Devalaraja-Narashimha, Michael P. Rosconi, Erica A. Pyles

**Affiliations:** ^1^ Product Biochemistry, Regeneron Pharmaceuticals, Inc., Tarrytown, New York, USA, regeneron.com; ^2^ Analytical Research and Development, Merck and Co., Inc., Rahway, New Jersey, USA; ^3^ Cardiovascular and Renal Research, Regeneron Pharmaceuticals, Inc., Tarrytown, New York, USA, regeneron.com

## Abstract

Therapeutic monoclonal antibodies (mAbs) remain an important segment of the biopharmaceutical industry, delivering highly effective and targeted responses against human diseases. The immune complexes formed between a therapeutic mAb and its target antigens may affect efficacy, clearance (pharmacokinetics), and immunogenicity. As such, immune complexes represent an important biophysical property to characterize during drug product screening and development. In this study, we leverage asymmetric flow field‐flow fractionation coupled with multiangle light scattering (A4F‐MALS) to evaluate the size distribution of complexes formed between a panel of therapeutic mAbs directed against a common target antigen. The inclusion of this analysis enables important insights that can guide the screening of therapeutic candidates during lead candidate assessments, as well as risk mitigation to help better inform clinical design strategy.

## 1. Introduction

Therapeutic monoclonal antibodies (mAbs) are amongst the most active areas of research and development in the pharmaceutical industry for many clinical disease indications. The advantages of therapeutic mAbs include their high specificity, long half‐life, and low toxicity. When therapeutic mAbs bind with their targets, they form a drug‐target complex that, if shown to elicit an immune response, is termed an immune complex. The immune complex can induce adverse reactions, including type III hypersensitivity reactions that may cause inflammatory injury. In type III hypersensitivity reactions, immune complexes formed from antigen‐antibody higher‐order complexes can be deposited in various tissues, including blood vessels, kidneys, and joints [1, 2]. These deposits may activate the complement system, which increases vascular permeability and leads to the recruitment of inflammatory cells, which trigger an inflammatory response leading to tissue damage. Additionally, immune complexes can bind to Fcγ receptors on immune cells, which enables the release of inflammatory mediators and triggers an inflammatory response [3]. Characterization of immune complex‐dependent adverse reactions in preclinical studies is therefore of high interest to the pharmaceutical industry [4].

Immune complexes are typically composed of a lattice‐like network of noncovalently bound antibody and antigen molecules. This lattice is dependent on antibody isotype and conformation, as well as local antigen and antibody concentrations, their stoichiometric ratio and valence (number of repeating units/epitopes), and their binding affinity [5]. For clinical trials involving multiple antibody therapeutics targeting the same target at different epitopes, such as coformulated/coadministered products and/or clinical drug switch studies, there is a greater risk of formation of more complicated multivalent immune complexes. One recent example in the treatment of paroxysmal nocturnal hemoglobinuria involved a clinical drug switch study from a comparator mAb, the current standard of care, to crovalimab [6]. Compared to the comparator mAb‐naïve cohort, two patients in the drug switch cohort developed skin reactions that were considered related to treatment. This outcome was attributed to the potential formation of drug‐target‐drug immune complexes comprising human complement component 5 (hC5) and both anti‐hC5 antibodies [6], given that the comparator mAb and crovalimab are able to engage hC5 simultaneously and noncompetitively at distinct epitopes.

**Figure 1:**
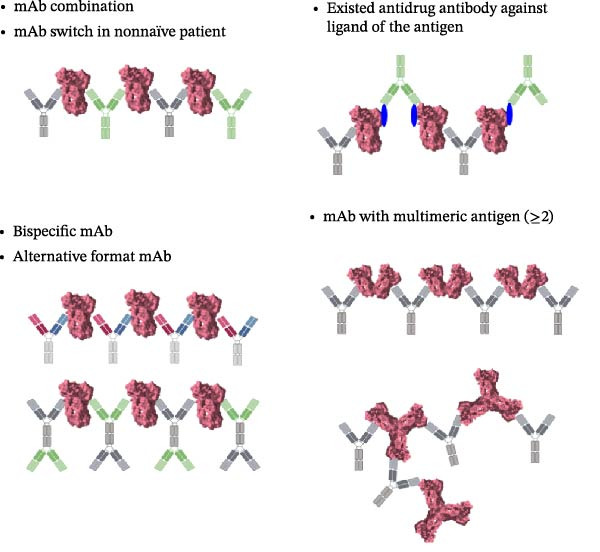
Schematic overview of the scenarios involving higher‐order complex formation between an antibody and antigen.

The size of immune complexes has also been implicated in the occurrence and severity of adverse events. More specifically, studies reported severe infusion reactions to infliximab for the treatment of rheumatoid arthritis or Crohn’s disease. Infusion reactions were correlated with lower clinical response durations and/or the detection of antidrug antibodies, which are generated in response to the therapeutic drug itself and can also contribute to the formation of large immune complexes [7, 8]. Indeed, immune complexes exceeding ~1000 kDa were only detected in the serum samples of nonresponsive patients who exhibited nonnegligible levels of anti‐infliximab antibodies, including one patient whose severe infusion reaction required withdrawal from treatment, compared to two patients whose serum samples exhibited no signs of immune complexes [7]. These results suggested that large immune complexes were more likely to be associated with adverse events and that the size of immune complexes might serve as a marker for immune complex‐dependent reactions [9, 10]. Therefore, the potential formation and size distribution of drug‐target complexes should be evaluated when developing antibody therapeutics. This includes various scenarios such as coadministered or coformulated antibody cocktail therapy, drug switching in nonnaïve patients, the presence of pre‐existing antidrug antibodies against the ligand of the antigen, the use of bispecific or alternative mAbs, or the development of bivalent antibodies against multimeric antigens (Figure 1).

The evaluation of the size distribution of drug‐target complexes could be achieved using a variety of traditional biophysical tools. Size‐exclusion chromatography (SEC) is a widely used separation technique that separates particles based on size as they pass through a gel‐filtration column. When coupled with multiangle light scattering (MALS), SEC‐MALS can define the stoichiometry of discrete mAb‐antigen complexes in solution [11]. However, the resolving power of SEC is limited by gel pore size and nonspecific adsorption to the column matrix. Moreover, the packed particle bed gives rise to potential shear forces, which can degrade sensitive samples [12]. Asymmetric flow field‐flow fractionation (A4F) represents an orthogonal native size characterization method that overcomes some of the key challenges described for SEC by direct analysis of samples in the matrix of interest [13, 14]. In brief, A4F is a gentle separation technique that utilizes perpendicular flow forces to fractionate analytes within a vertically thin channel based on differences in their diffusion coefficients, which, in turn, are related to the size and shape of the fractionating particle. However, unlike SEC, A4F does not rely on a stationary matrix to perform the separation and has a very broad, customizable resolution range (from 1 nm to 10 μm). Fractionation of samples by A4F eliminates shear forces and allows for the detection of large aggregates and/or particles that would not necessarily be observed by SEC. Taken together, these distinct advantages render A4F a versatile tool for analyzing the molar mass and size distribution of complicated multivalent drug‐target complex networks when coupled to MALS detection.

In this study, with hC5 as an example, we developed and leveraged a versatile A4F‐MALS method to enable the characterization of antibody‐ligand complexes during early candidate screening and preclinical development. In a simulated drug switch, we also demonstrated that there is likely to be a low risk of higher‐order complex formation in nonnaïve patients switching from the comparator mAb to an anti‐C5 bispecific antibody (bsAb). This study enables important insights that can support therapeutic candidate screening, as well as support risk assessments for drug switch studies in clinical design.

## 2. Materials and Methods

### 2.1. Reagents

All mAbs and bsAbs (mAb‐A, comparator mAb, mAb‐B, mAb‐C, mAb‐D, mAb‐E, mAb‐F, mAb‐G, and bsAb) used in this experiment were expressed in Chinese hamster ovary cells and produced at Regeneron Pharmaceuticals, Inc., according to the protocol defined in Haber et al. [15]. Antigen hC5 was purchased from Sigma Cat# 204888‐250UG, Millipore Sigma).

#### 2.1.1. Sample Preparation

Defined amounts of antibodies or antibody combinations were combined with hC5 and diluted in 1 X DPBS, pH 7.4, to yield the various molar ratios (mAb:hC5 or mAb‐X:mAb‐Y:hC5). All samples were equilibrated at ambient temperature for 2 h and maintained unfiltered at 4°C in the autosampler chamber prior to the analysis. Individual samples of antibodies and hC5 were prepared separately and included as controls under these conditions.

#### 2.1.2. A4F Separation and MALS Method

The A4F measurements were performed using a Wyatt Technology small separation channel (18 cm) equipped with a 350‐μm spacer with 10 kDa molecular weight cutoff regenerated cellulose (Wyatt); an Eclipse 3+ separation system (Wyatt Technology); an Agilent 1200 HPLC series isocratic pump, autosampler, degasser, and UV detector (Agilent Technologies, Palo Alto, California); and a Wyatt Technology DAWN MALS and Optilab T‐rEX differential refractometer detector. The sample concentration was measured at 215 nm via UV detection. The MALS detector was used for the determination of the average molecular weight of the complex.

The detector flow was maintained at 1.0 mL/min for the duration of the run. Samples were injected onto the channel at 0.38 mL/min and focused for 2 min using a focus flow rate of 1.5 mL/min to allow all sample particles to focus prior to fractionation. The separation step consisted of a linear gradient of the cross‐flow from 3.0 mL/min to 0.0 mL/min over 45 min before holding the cross‐flow for a further 5 min at 0.0 mL/min to allow any large particles to elute. The mobile phase for all experiments was 10 mM sodium phosphate and 500 mM sodium chloride, pH 7.0 ± 0.1. BSA was injected as a system suitability control. About 4 μg of each control sample was injected onto the channel, and 7 μg of each complex sample was injected.

### 2.2. A4F‐MALS Data Analysis

Data were analyzed using Astra software (Version 7.3.1, Wyatt Technology). The data were fit to Equation (1), which relates the excess scattered light to the solute concentration and weight‐average molar mass, Mw.

(1) K∗cRθ,c=1MwPθ+2A2c,

where *c* is the solute concentration, *R*(*θ*,*c*) is the excess Rayleigh ratio from the solute as a function of scattering angle and concentration, *Mw* is the weight‐average molar mass, *P*(*θ*) is the angular dependence of scattered light (~1 for particles with radius of gyration <50 nm), *A*_2_ is the second virial coefficient in the expansion of osmotic pressure (which can be neglected since measurements are performed on dilute solutions), and 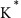 is defined by Equation (2).

(2) K∗=4π2n02NAλ04dndc2,

where *n*_0_ represents the solvent refractive index, *N*_*A*_ is Avogadro’s number, *λ*_0_ is the wavelength of the incident light in a vacuum, and *dn/dc* represents the specific refractive index increment for the solute.

The normalization coefficients for the light scattering detectors, interdetector delay volume, and band‐broadening terms were calculated from the BSA chromatograms. These values were applied to the data files collected for mAb and complex samples to correct for these terms.

The *dn/dc* value of a protein is dependent on the salt concentration. A corrected *dn/dc* value of 0.182 mL/g was used to account for the high salt content in the mobile phase. The UV extinction coefficient of the antibody and antigen controls was calculated at 215 nm using their respective amino acid sequences. Following by the protein conjugate analysis of the controls, the *dn/dc* and 215 nm UV extinction coefficients of antibodies and hC5 were calculated, and weighted averages were calculated for the analysis of the prepared complex samples.

## 3. Results and Discussions

### 3.1. A4F‐MALS Size Distribution Analysis Can be Applied During Candidate Selection as a Tool to Screen Combination Therapies

Blockade of the target is an important strategy in treating and controlling these disease conditions. Both published findings by Harder et al. [16] and internal in vivo studies (not shown) suggested that adding a second blocking antibody to the comparator mAb can further suppress the hC5 alternative pathway compared to monotherapy [16]. However, combination therapy comprised of antibodies binding noncompetitively to different epitopes of the same target presents the risk of inducing higher order drug‐target‐drug complexes. Previously, we used A4F‐MALS to characterize the size distribution of mAb‐A:in‐house comparator mAb:C5 complex with the molar ratio that was expected in vivo at the time of the initial dose switch in the clinic [17]. Here, the A4F‐MALS method was used to characterize complexes formed between therapeutic mAbs and target antigen, and to compare the size of drug‐target complexes between different combo antibodies and the antigen.

**Figure 2:**
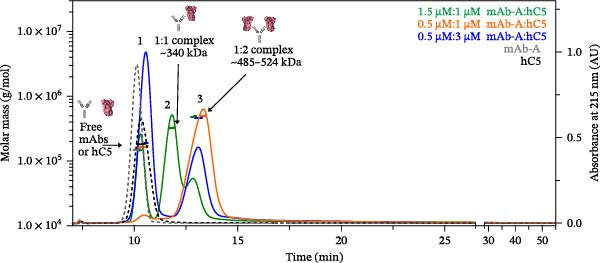
A4F‐MALS fractograms of mAb‐A and hC5 samples. Overlaid A4F‐MALS fractograms of mAb‐A:hC5 complexes prepared at various molar ratios. Gray dashed trace, free intact mAb‐A; black dashed trace, free hC5; green trace, 3:2 mAb‐A:hC5 complex; orange trace, 1:2 mAb‐A:hC5 complex; and blue trace, 1:6 mAb‐A:hC5 complex. The right *Y*‐axis is normalized intensity of the absorbance signal at 215 nm, whereas the left *Y*‐axis is molar mass determined by MALS. Molar mass is shown as a straight line in color corresponding to the fractogram trace. Experiments were performed independently twice with comparable results; for consistency and clarity, data from a representative experiment are shown. AU, absorbance unit.

As a control, mAb‐A and hC5 were mixed at 3:2, 1:2, and 1:6 molar ratios to test stoichiometric ratios (Figure 2). The annotated peak numbers and molar masses correspond to partially resolved mAb‐A:C5 complexes (peaks 2 and 3) as well as free mAb‐A and free hC5 (peak 1). The measured molar masses of coeluting free intact mAb‐A and free hC5 in peak 1 were ~153.9 kDa and 191.8 kDa, respectively. Based on the calculated average molar masses of ~340.2 kDa and 510.8 kDa, peaks 2 and 3 most likely correspond to 1:1 and 1:2 mAb‐A:hC5 complexes, respectively (Table 1). Under conditions of excess mAb‐A relative to hC5, the 1:1 mAb‐A:C5 complex was more abundant than the 1:2 complex. At equimolar ratios of antibody binding sites to antigen (molar ratio of 1:2 mAb‐A:hC5), the 1:2 complex form was present at 94.3%, indicating that mAb‐A possesses two equivalent binding sites for hC5. When hC5 was present in excess, the predominant bound species was the 1:2 mAb‐A:C5 complex, with minimal detectable levels of free mAb‐A or 1:1 complex (Table 1). Additionally, no higher molecular weight complexes were detected for any sample under any of the tested stoichiometric ratios, indicating that mAb‐A binds to hC5 without higher‐order multimerization.

**Table 1:** Molar masses and relative abundance of mAb‐A complexes with hC5.

(mAb‐A) (µM)	(hC5) (µM)	Molar ratio mAb‐A:hC5	Peak 1		Peak 2		Peak 3	
			Free mAb/hC5		mAb_1_:hC5_1_ complex		mAb_1_:hC5_2_ complex	
			Mean MW (kDa)^a^	Mean peak area (%)^b^	Mean MW (kDa)^a^	Mean peak area (%)^b^	Mean MW (kDa)^a^	Mean peak area (%)^b^
6.7	0	1:0	153.9 (0.9)^a^	100 (0.0)^b^	ND	ND	ND	ND
0	5.3	0:1	191.8 (0.5)^a^	100 (0.0)^b^	ND	ND	ND	ND
1.5	1	3:2	153.9	27.5	340.2	50.3	510.8	22.2
0.5	1	1:2	172.6	5.7	ND	ND	524.4	94.3
0.5	3	1:6	197.6	58.0	ND	ND	485.0	42.0

Abbreviation: ND, not detected.

^a^Mean of the weight‐average molar mass (kDa) is reported with standard deviation in parentheses. Reported values were obtained from duplicate injections of a single sample preparation.

^b^Peak area percentages were calculated from the UV absorbance detector for each sample injected onto the column. Each sample was injected in duplicate, and the average peak area percentages are reported with standard deviation in parentheses.

Combination therapy involving antibodies that bind noncompetitively to different epitopes on the same target presents the risk of forming higher‐order drug‐target‐drug complexes. The size of these complexes is affected by the molar ratio of the combined antibodies and antigens, with the largest complexes typically forming when these components are at or near equal molar ratios. Therefore, we used A4F‐MALS to assess the propensity of eight anti‐hC5 combinations to form higher‐order, heteromeric complexes at equimolar ratios of 0.5:0.5:1 molar ratio of mAb‐X:mAb‐Y:hC5.

**Figure 3:**
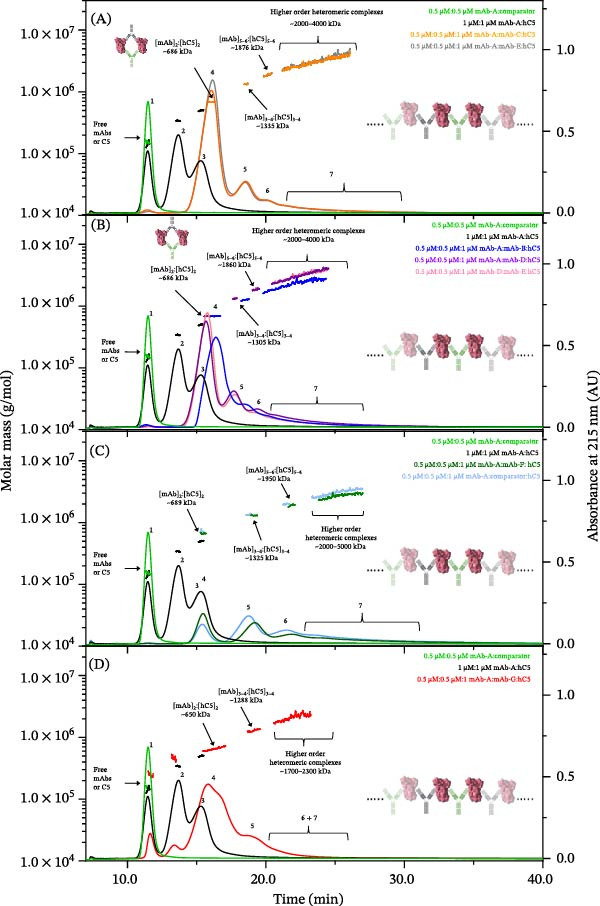
A4F‐MALS fractograms of combination mAbs and hC5 complexes. Overlaid A4F‐MALS fractograms of free mAb combination, 1:1 mAb‐A:hC5, and 1:1:2 mAb combination:hC5. (A) Green trace, free mAb‐A and comparator combination; black trace, 1:1 mAb‐A:hC5 complex; orange trace, 1:1:2 mAb‐A:mAb‐C:hC5; gray trace, 1:1:2 mAb‐A:mAb‐E:hC5. (B) Green trace, free mAb‐A and comparator combination; black trace, 1:1 mAb‐A:hC5 complex; blue trace, 1:1:2 mAb‐A:mAb‐B:hC5; purple trace: 1:1:2 mAb‐A:mAb‐D:hC5; pink trace, 1:1:2 mAb‐D:mAb‐E:hC5. (C) Green trace, free mAb‐A and comparator combination; black trace, 1:1 mAb‐A:hC5 complex; dark green trace, 1:1:2 mAb‐A:mAb‐F:hC5; light blue trace: 1:1:2 mAb‐A:comparator:hC5. (D) Green trace, free mAb‐A and comparator combination; black trace, 1:1 mAb‐A:hC5 complex; red trace, 1:1:2 mAb‐A:mAb‐G:hC5. The right *Y*‐axis is normalized intensity of the absorbance signal at 215 nm, whereas the left *Y*‐axis is molar mass determined by MALS. Molar mass is shown as a straight line in color corresponding to the fractogram trace. Experiments were performed independently twice with comparable results; for consistency and clarity, data from a representative experiment are shown.

The stoichiometries of the resulting complexes from all samples analyzed are presented in Figure 3 and Table 2. Peak 1 represents free intact mAb or hC5, and peak 2 and 3 represent mAb_1_:hC5_1_ and mAb_1_:hC5_2_ complexes, respectively. Overall, all mAb combinations exhibited the ability to form heteromeric complexes with hC5, with five combinations (mAb‐A/mAb‐C, mAb‐A/mAb‐E, mAb‐A/mAb‐B, mAb‐A/mAb‐D, and mAb‐D/mAb‐E) favoring a smaller discrete species (Peak 4) and minor amounts of larger, discrete complexes (peak 5) (Figure 3A,B). Based on the molar masses determined for coeluting free hC5 and free mAb (~195 kDa and ~150 kDa, respectively), the theoretical molar masses of mAb_2_:hC5_2_, mAb_4_:hC5_4_and mAb_6_:hC5_6_ complexes are predicted to be ~690 kDa, 1380 kDa, and 2070 kDa, respectively. Therefore, based on calculated average molar masses of ~686 kDa, 1320 kDa, and 1868 kDa, peaks 4, 5, and 6 most likely correspond to discrete populations of mAb_2_:hC5_2_, mAb_3-4_:hC5_3-4_, and mAb_5-6_:hC5_5-6_ complexes, respectively (Table 2). Peak 7 was very broad and comprises a heterogeneous population of very large (≥1700 kDa), extended antibody‐antigen lattices, termed “paper‐dolling” (Table 2). Only low levels (7%–16%) of paper‐dolling were observed for these five combinations (Figure 3).

**Table 2:** Approximate molar mass and retention time of anti‐hC5 mAb combinations with hC5 complexes at 0.5:0.5:1 molar ratio.

Sample	Peak 1		Peak 2		Peak 3			
	Free mAb		mAb_1_:hC5_1_ complex		mAb_1_:hC5_2_ complex			
	*R*_t_ (min)	*M*_w_ (kDa)	*R*_t_ (min)	*M*_w_ (kDa)	*R*_t_ (min)	*M*_w_ (kDa)		
mAb‐A:hC5	11.5	144.1	13.7	341.1	15.3	498.7		
mAb‐A:mAb‐B:hC5	ND	ND	ND	ND	ND	ND		
mAb‐A:mAb‐C:hC5	ND	ND	ND	ND	ND	ND		
mAb‐A:mAb‐D:hC5	ND	ND	ND	ND	ND	ND		
mAb‐A:mAb‐E:hC5	ND	ND	ND	ND	ND	ND		
mAb‐A:mAb‐F:hC5	ND	ND	ND	ND	ND	ND		
mAb‐A:mAb‐G:hC5	ND	ND	ND	ND	ND	ND		
mAb‐D:mAb‐E:hC5	ND	ND	ND	ND	ND	ND		
mAb‐A:comparator mAb:hC5	ND	ND	ND	ND	ND	ND		

Sample	Peak 4		Peak 5		Peak 6		Peak 7	
	mAb_2_:hC5_2_ complex		mAb_3-4_:hC5_3-4_ complex		mAb_5-6_:hC5_5-6_ complex		Higher order heteromeric complexes (mAb_≥7_: hC5_≥7_)	
	*R*_t_ (min)	*M*_w_ (kDa)	*R*_t_ (min)	*M*_w_ (kDa)	*R*_t_ (min)	*M*_w_ (kDa)	*R*_t_ (min)	*M*_w_ (kDa)
mAb‐A:hC5	ND	ND	ND	ND	ND	ND	ND	ND
mAb‐A:mAb‐B:hC5	16.4	684.7	18.4	1261.8	ND	ND	20.2	~1700–2700
mAb‐A:mAb‐C:hC5	16.0	684.4	18.5	1342.4	20.1	1876.0	21.5	~2250–3560
mAb‐A:mAb‐D:hC5	15.7	685.9	17.7	1319.8	19.4	1849.8	20.6	~2300–3800
mAb‐A:mAb‐E:hC5	16.1	687.7	18.5	1327.4	20.1	1865.6	21.5	~2380–4250
mAb‐A:mAb‐F:hC5	15.5	664.8	19.2	1304.3	21.9	1901.1	23.6	~2300–4100
mAb‐A:mAb‐G:hC5	15.9	649.6	19.1	1288.2	ND	ND	20.6	~1700–2300
mAb‐D:mAb‐E:hC5	15.8	687.7	17.8	1333.8	19.3	1871.6	20.6	~2300–3600
mAb‐A:comparator mAb:hC5	15.4	713.7	18.8	1346.1	21.5	2001.9	23.6	~2400–5000

Abbreviations: kDa, kilodaltons; min, minutes; *M*_w_, weight‐average molar mass; ND, not detected; *R*_t_, retention time.

The mAb‐A/mAb‐B combination formed similar stoichiometry and size distributions of complexes with hC5 based on molar mass, but the mAb‐A/mAb‐B combination eluted later and with a broader, less‐resolved distribution of higher‐order complexes (Figure 3A,B). This shift in retention time may reflect antibody‐specific differences in shape or orientation, particularly for this combination.

In contrast, combinations of mAb‐A with mAb‐F and comparator mAb (Figure 3C,D) favored fractogram peaks 5 (~1325 kDa) and peak 6 (~1950 kDa), representing larger, more heterogeneous complexes with hC5. A higher degree of paper‐dolling (peak 7) was also observed for these combinations (Figure 3C and Table 2). Combinations of mAb‐A and mAb‐G (Figure 3D) formed heteromeric complexes with mAb_2_:hC5_2_ complexes as the predominant species (peak 4). Complexes containing 3–4 molecules of mAb bound to 3–4 molecules of hC5 antigen (peak 5) and very large, heterogeneous, extended antibody‐antigen lattices (>1700 kDa; peaks 6 and 7) were also minimally observed. Compared to the other combinations, mAb‐A/mAb‐G displayed a reduced tendency to form heteromeric complexes with hC5, as evidenced by the presence of free mAb and mAb_1_:hC5_1_ homomeric complexes in this sample (peaks 1 and 2). This result suggests that the binding of one mAb influences the affinity (and/or off‐rate) or changes local conformation, thereby affecting the binding of another anti‐hC5 mAb. Alternatively, this may indicate reduced stability of the heteromeric complexes in this sample during the fractionation process compared to that of the other combinations tested. Finally, the predominant complex observed in the fractogram of the mAb‐A/mAb‐G combination was mAb_2_:hC5_2_.

In this mAb combination screening, some mAb combinations (Figure 3C) formed higher‐order complexes, likely driven by the epitope geometry of hC5 and mAbs, whereas other mAb combinations can cobind hC5 without extended lattice formation, yielding defined stoichiometries and mitigating higher‐order species (Figure 3A,B). This result shows that an extended higher order complex is not a universal feature of mAb combination therapies; whether a second mAb can be accommodated will be dependent on epitope spacing and binding geometry.

### 3.2. A4F‐MALS Size Distribution Analysis Was Used to Support the Development of an Anti‐hC5 bsAb

**Figure 4:**
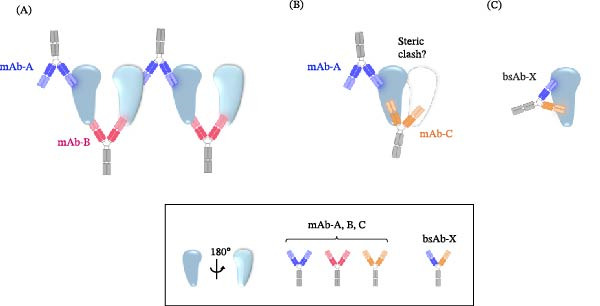
The formation of higher‐order drug‐target complexes may be influenced by location and distance between epitopes, as well as the angle of engagement. (A) In this simple scenario, mAb‐A and mAb‐B are assumed to bind their common ligand at distal, noncompeting epitopes on opposing interfaces, enabling the paper‐dolling effect when propagated in three‐dimensional space. (B) If mAb‐A and mAb‐C engage the ligand more proximally and on the same interface, this close interaction may result in potential steric clashes, which would prevent recruitment of additional C5 molecules and reduce the paper‐dolling effect. (C) The design of a bispecific antibody, which binds multiple epitopes on the ligand, and with sufficient affinity to displace the comparator mAb, may be a viable option to mitigate risks incurred during clinical drug‐switch studies.

Differences in the propensity of the various antibody combinations to form large, heterogenous, mAb:ligand complexes are likely associated with the location of each unique epitope relative to the other on the three‐dimensional surface of the antigen. One can postulate that the formation of large, extended drug‐target complexes would require the two unique epitopes for the individual mAbs to be located more distally from one another, whereas more closely located epitopes may hinder or prevent extended complexes from forming due to steric clashes (Figure 4). One engineering approach that may mitigate the formation of extended drug‐target complexes while maintaining the potential advantage of occupying multiple epitopes on a single antigen is to combine the individual variable regions of the previously identified experimental mAb combination, which displayed a lower propensity to form extended drug‐target complexes, into a single bispecific mAb.

**Figure 5:**
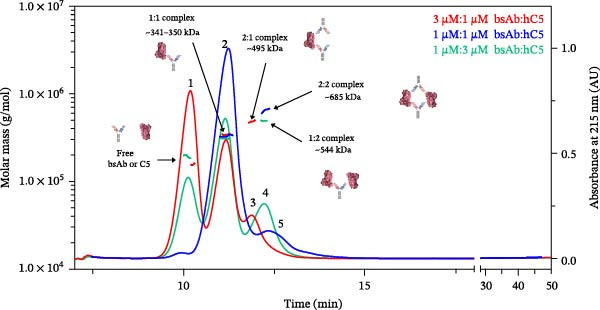
A4F‐MALS fractograms of bsAb and hC5 complexes. Overlaid A4F‐MALS fractograms of bsAb:hC5 complexes. Red trace, 3:1 bsAb:hC5 complex; blue trace, 1:1 bsAb:hC5 complex; and green trace, 1:3 bsAb:hC5 complex. The right *Y*‐axis is normalized intensity of the absorbance signal at 215 nm, whereas the left *Y*‐axis is molar mass determined by MALS. Molar mass is shown as a straight line in color corresponding to the fractogram trace. Experiments were performed independently twice with comparable results; for consistency and clarity, data from a representative experiment are shown.

The bispecific anti‐hC5 mAb (bsAb) was engineered based on the mAb‐D/mAb‐E combination, with each Fab arm binding a unique epitope on hC5. When mixed at molar ratios of 3:1, 1:1, and 1:3, bsAb predominantly forms a discrete complex with hC5 (peak 2; Figure 5). Importantly, no additional higher‐order complexes were observed, indicating that bsAb does not promote paper‐dolling with hC5. Based on the molar masses determined for free intact bsAb (~150 kDa) and free hC5, the theoretical molar masses for 1:1, 2:1, 1:2, and 2:2 complexes are predicted to be ~345, 497, 538, and 690 kDa. Therefore, fractogram peak 2 most likely corresponds to 1:1 intact bsAb:hC5 complexes based on a calculated average molar mass of ~345 kDa (Table 3). For all three molar ratios evaluated, the 1:1 bsAb:hC5 complex appears to be the predominant bound species (peak 2, ~83%), indicating that the bsAb successfully mitigates the formation of extended complexes likely by forming an avidity‐driven complex and/or by reducing the favorability of having multiple bsAbs bound to the same antigen molecule. Only minor amounts of additional discrete complexes consistent with 2:1 (peak 3, ~495 kDa, 14%), 1:2 (peak 4, ~544 kDa, ~23%), and 2:2 (peak 5, ~685 kDa, ~17%) bsAb:hC5 complexes were detected primarily when either the bsAb or hC5 was present in excess of the other.

**Table 3:** Approximate molar mass and retention time of bispecific bsAb with hC5 complexes.

Sample	Molar ratio (µM:µM)	Peak 1		Peak 2		Peak 3		Peak 4		Peak 5	
		Free mAb or free C5		bsAb_1_:hC5_1_ complex		bsAb_2_:hC5_1_ complex		bsAb_1_:hC5_2_ complex		bsAb_2_:hC5_2_ complex	
		*R*_t_ (min)	*M*_w_ (kDa)	*R*_t_ (min)	*M*_w_ (kDa)	*R*_t_ (min)	*M*_w_ (kDa)	*R*_t_ (min)	*M*_w_ (kDa)	*R*_t_ (min)	*M*_w_ (kDa)
bsAb:hC5	3:1	10.7	164.0	11.2	359.3	11.9	506.5	ND	ND	ND	ND
bsAb:hC5	1:1	ND	ND	11.2	347.2	ND	ND	ND	ND	12.3	681.3
bsAb:hC5	1:3	10.1	218.9	11.1	350.9	ND	ND	12.2	559.5	ND	ND

Abbreviations: kDa, kilodaltons; min, minutes; *M*_w_, weight‐average molar mass; ND, not detected; *R*_t_, retention time.

These results contrast with the complexes formed between the mixture of the individual parental mAbs (mAb‐D and mAb‐E), which exhibited a low propensity to form higher order heteromeric complexes containing multiple molecules of hC5 and no evidence of a stable 1:1 or 2:1 mAb:hC5 complex detected (Figure 3B). Taken together, these data demonstrate that the engineered bsAb successfully mitigates the formation of extended complexes by increasing the favorability of forming a stable 1:1 complex with a single molecule of hC5 in comparison to the mixture of parental mAbs from which it was derived. Furthermore, this indicates that epitope location, valency, and avidity may influence the binding mode and/or orientation of each Fab to the hC5 epitope and hence impact complex size and stoichiometry.

### 3.3. Applications of A4F‐MALS to Assess the Risk of Drug Switching in Nonnaïve Patient Populations

If a new anti‐hC5 antibody shows promise as a therapeutic option for patients with rare genetic variants of C5, it could serve as an alternative for those currently treated with a comparator mAb. However, combining antibodies that bind to unique epitopes on a soluble antigen can lead to the formation of higher‐order protein complexes, potentially causing type‐III hypersensitivity reactions. Previous studies using A4F‐MALS to examine the size of mAb‐A:in‐house comparator mAb:hC5 complexes suggest that while in‐house comparator mAb and mAb‐A can form very large, heterogeneous higher‐order complexes with hC5 at or near equimolar ratio, the formation of heteromeric complexes is minimal at concentrations expected in vivo during the initial dose switch. After establishing bsAb as a viable candidate targeting hC5, it became essential to assess its tendency to form multivalent higher‐order complexes, which could pose immunogenic risks during a drug switch clinical trial for nonnaïve patients with rare blood disorders.

**Figure 6:**
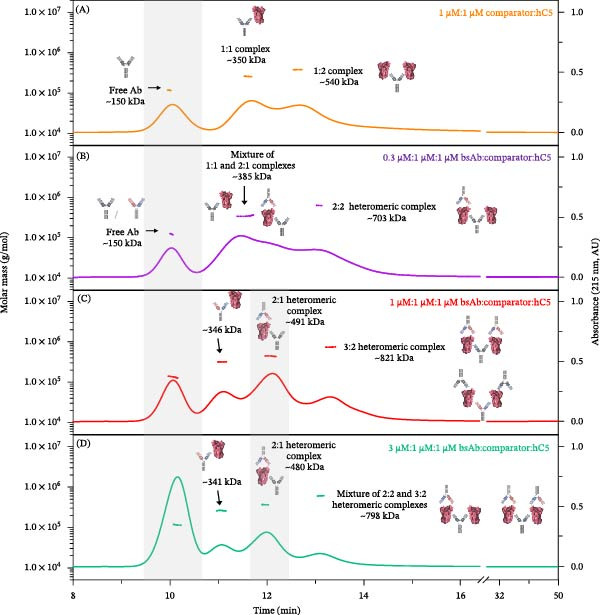
A4F‐MALS fractograms of bsAb, in‐house comparator, and hC5 complexes. A4F‐MALS fractograms of comparator:hC5 complex and bsAb:comparator:hC5 complexes. (A) Orange trace, 1:1 comparator:hC5. (B) Purple trace, 0.3:1:1 bsAb:comparator:hC5. (C) Red trace, 1:1:1 bsAb:comparator:hC5. (D) Green trace, 3:1:1 bsAb:comparator:hC5. The right *Y*‐axis is normalized intensity of the absorbance signal at 215 nm, whereas the left *Y*‐axis is molar mass determined by MALS. Molar mass is shown as straight line in color corresponding to the fractogram trace. Experiments were performed independently twice with comparable results; for consistency and clarity, data from a representative experiment are shown.

To simulate in vitro conditions that may be used in a clinical drug switch study, bsAb was added to preformed complexes of in house comparator mAb:hC5 complex, with the comparator mAb representing the current standard of care. Representative A4F‐MALS fractograms corresponding to various molar ratio mixtures of bsAb, the comparator mAb, and hC5 are overlaid in Figure 6. Overall, when bsAb was added to preformed comparator mAb:hC5 complexes, stable, discrete heteromeric complexes were observed under various conditions with a maximum stoichiometric ratio of bsAb_3_:hC5_2_. No higher‐molecular weight complexes were detected at any molar ratio. This data suggest that there is low risk of higher‐order complex formation with bsAb treatment in nonnaïve patients previously treated with or switching directly from a comparator mAb.

## 4. Discussion

The A4F‐MALS method represents a robust and innovative approach for assessing the stoichiometry of antibody‐antigen complexes. This in vitro assay enhances the understanding of complex formation resulting from therapeutic antibody administration in patients and offers valuable insights to support the clinical study design. This method offers the flexibility to adjust the separation range to manage the wide size distribution of samples, accommodates samples with a broad concentration range, and provides high‐throughput characterization of complexes ranging from 1 nm to several microns hydrodynamic radius. Although the setup and operation of A4F‐MALS systems require specialized equipment and expertise, the insights that it provides are invaluable to understanding complex formation of antibodies binding to antigens. Complementary orthogonal biophysical techniques such as analytical ultracentrifugation (AUC) and mass photometry offer unique advantages. AUC offers high‐resolution stoichiometric data under native conditions but is low‐throughput. Mass photometry enables rapid, precise stoichiometry determination at low concentrations but is constrained by its detection threshold (~30 kDa) and challenges with weak biomolecular interactions. However, the A4F‐MALS approach to evaluate stoichiometry of complexes offers significant advantages over AUC and mass photometry. These advantages include its ability to resolve a broader distribution of size and concentration ranges, along with its high‐throughput capacity, thereby establishing it as a pivotal tool in therapeutic antibody research, allowing for extensive screening of antibody candidates or alternative format mAbs, differentiation of antibody‐antigen systems, and detection of higher order drug‐target complexes. This information can be used to assess the potential immunogenicity risks associated with therapeutic candidates. By understanding the stoichiometry and size distribution of antibody‐antigen complexes, researchers can also design dosing regimens that minimize the risk of forming large, immunogenic complexes. This is particularly important for drug switching in nonnaïve patient populations, where the presence of pre‐existing antibodies can influence the formation of drug‐target complexes. Combining stoichiometry analysis with orthogonal biological assays, such as complement activation assays, can provide a more comprehensive understanding of the potential immunogenicity risks. This integrated approach can help identify candidates with lower immunogenicity risks and support the development of safer and more effective therapies.

## 5. Conclusion

The A4F‐MALS method is a powerful and versatile tool for evaluating the stoichiometry and size distribution of antibody‐antigen complexes, offering critical insights into therapeutic antibody research. It can handle a wide range of sample sizes and concentrations with high‐throughput capabilities. By providing detailed information on complex formation, this method aids in assessing immunogenicity risks, optimizing dosing regimens, and supporting clinical study designs, particularly for drug switching in nonnaïve patient populations. Integrating A4F‐MALS with orthogonal assays further enhances its utility, enabling the development of safer and more effective therapies.

## Funding

No funding was received for this manuscript.

## Conflicts of Interest

The authors declare no conflicts of interest.

## Data Availability

The data that support the findings of this study are available from the corresponding author upon reasonable request.

## References

[1] Uzzaman A. and Cho S. H. , Chapter 28: Classification of Hypersensitivity Reactions, Allergy and Asthma Proceedings. (2012) 33, no. 3, 96–99, 10.2500/aap.2012.33.3561, 2-s2.0-84863206402. 22794701

[2] Torres J. S. S. and Annamaraju P. , Type III Hypersensitivity Reaction, 2025, StatPearls Publishing. 32644548

[3] Nimmerjahn F. and Ravetch J. V. , Fcγ Receptors as Regulators of Immune Responses, Nature Reviews Immunology. (2008) 8, no. 1, 34–47, 10.1038/nri2206, 2-s2.0-37549036732. 18064051

[4] Fichter M. , Richter G. , Bepperling A. , and Wassmann P. , Pre-Clinical In-Vitro Studies on Parameters Governing Immune Complex Formation, Pharmaceutics. (2022) 14, no. 6, 10.3390/pharmaceutics14061254, 1254. 35745826 PMC9227392

[5] Mannik M. , Physicochemical and Functional Relationships of Immune Complexes, Journal of Investigative Dermatology. (1980) 74, no. 5, 333–338, 10.1111/1523-1747.ep12543582, 2-s2.0-0018963465. 6446581

[6] Röth A. , Nishimura J. , and Nagy Z. , et al.The Complement C5 Inhibitor Crovalimab in Paroxysmal Nocturnal Hemoglobinuria, Blood. (2020) 135, no. 12, 912–920, 10.1182/blood.2019003399. 31978221 PMC7082616

[7] van der Laken C. J. , Voskuyl A. E. , and Roos J. C. , et al.Imaging and Serum Analysis of Immune Complex Formation of Radiolabelled Infliximab and Anti-Infliximab in Responders and Non-Responders to Therapy for Rheumatoid Arthritis, Annals of the Rheumatic Diseases. (2007) 66, no. 2, 253–256, 10.1136/ard.2006.057406, 2-s2.0-33846855387. 16793840 PMC1798492

[8] Lichtenstein L. , Ron Y. , and Kivity S. , et al.Infliximab-Related Infusion Reactions: Systematic Review, Journal of Crohn’s and Colitis. (2015) 9, no. 9, 806–815, 10.1093/ecco-jcc/jjv096, 2-s2.0-85018196509. PMC4558633 26092578

[9] Murdaca G. , Negrini S. , and Greco M. , et al.Immunogenicity of Infliximab and Adalimumab, Expert Opinion on Drug Safety. (2019) 18, no. 5, 343–345, 10.1080/14740338.2019.1602117, 2-s2.0-85064686749. 30938213

[10] Ungan D. , Be C. , and Baczyk P. , et al.IL-17A Complexes With Therapeutic Antibodies Exhibit Distinct Size Distributions, Potentially Contributing to Clinically Observed Immunogenicity, mAbs. (2025) 17, no. 1, 10.1080/19420862.2025.2575840, 2575840. 41111004 PMC12536631

[11] Castellanos M. M. , Snyder J. A. , and Lee M. , et al.Characterization of Monoclonal Antibody–Protein Antigen Complexes Using Small-Angle Scattering and Molecular Modeling, Antibodies. (2017) 6, no. 4, 10.3390/antib6040025, 25. 30364605 PMC6197476

[12] D’Atri V. , Imiołek M. , and Quinn C. , et al.Size Exclusion Chromatography of Biopharmaceutical Products: From Current Practices for Proteins to Emerging Trends for Viral Vectors, Nucleic Acids and Lipid Nanoparticles, Journal of Chromatography A. (2024) 1722, 10.1016/j.chroma.2024.464862, 464862. 38581978

[13] Fraunhofer W. and Winter G. , The Use of Asymmetrical Flow Field-Flow Fractionation in Pharmaceutics and Biopharmaceutics, European Journal of Pharmaceutics and Biopharmaceutics. (2004) 58, no. 2, 369–383, 10.1016/j.ejpb.2004.03.034, 2-s2.0-3843143950. 15296962

[14] Wahlund K.-G. , Flow Field-Flow Fractionation: Critical Overview, Journal of Chromatography A. (2013) 1287, 97–112, 10.1016/j.chroma.2013.02.028, 2-s2.0-84875682235. 23510956

[15] Haber L. , Olson K. , and Kelly M. P. , et al.Generation of T-Cell-Redirecting Bispecific Antibodies With Differentiated Profiles of Cytokine Release and Biodistribution by CD3 Affinity Tuning, Scientific Reports. (2021) 11, no. 1, 10.1038/s41598-021-93842-0, 14397. 34257348 PMC8277787

[16] Harder M. J. , Kuhn N. , and Schrezenmeier H. , et al.Incomplete Inhibition by Eculizumab: Mechanistic Evidence for Residual C5 Activity During Strong Complement Activation, Blood. (2017) 129, no. 8, 970–980, 10.1182/blood-2016-08-732800, 2-s2.0-85014887309. 28028023 PMC5324716

[17] Latuszek A. , Liu Y. , and Olsen O. , et al.Inhibition of Complement Pathway Activation With Pozelimab, A Fully Human Antibody to Complement Component C5, PLoS ONE. (2020) 15, e0231892. 32384086 10.1371/journal.pone.0231892 PMC7209288

